# Refining the mandibular osteoradionecrosis rat model by in vivo longitudinal µCT analysis

**DOI:** 10.1038/s41598-021-01229-y

**Published:** 2021-11-15

**Authors:** Morgane Dos Santos, Christelle Demarquay, Louis Ermeneux, Fazia Aberkane, Pauline Bléry, Pierre Weiss, Fabien Milliat, Noëlle Mathieu

**Affiliations:** 1 Human Health Department, IRSN, Institute for Radioprotection and Nuclear Safety, PSE-SANTE, SERAMED, LRMed, 92 262 Fontenay-aux-Roses, France; 2 CHU Nantes, INSERM, Regenerative Medicine and Skeleton, RMeS, UMR 1229, Université de Nantes, Oniris, 44000 Nantes, France; 3 IRSN, Institute of Radioprotection and Nuclear Safety, Human Health Department, PSE-SANTE, SERAMED, LRAcc, 92 262 Fontenay-aux-Roses, France

**Keywords:** Oral diseases, Biological physics

## Abstract

Osteoradionecrosis (ORN) is one of the most feared side effects of radiotherapy following cancers of the upper aero-digestive tract and leading to severe functional defects in patients. Today, our lack of knowledge about the physiopathology restricts the development of new treatments. In this study, we refined the ORN rat model and quantitatively studied the progression of the disease. We tested the impact of radiation doses from 20 to 40 Gy, delivered with incident 4MV X-ray beams on the left mandible of the inbred Lewis Rat. We used micro-computed tomography (µCT) to obtain in vivo images for longitudinal bone imaging and ex vivo images after animal perfusion with barium sulphate contrast agent for vessel imaging. We compared quantification methods by analyzing 3D images and 2D measurements to determine the most appropriate and precise method according to the degree of damage. We defined 25 Gy as the minimum irradiation dose combined with the median molar extraction necessary to develop non-regenerative bone necrosis. µCT image analyses were correlated with clinical and histological analyses. This refined model and accurate methods for bone and vessel quantification will improve our knowledge of the progression of ORN pathology and allow us to test the efficacy of new regenerative medicine procedures.

## Introduction

The treatment of squamous cell carcinomas of the upper aero-digestive tract (UAT) remains a major current health challenge. Treatment is based on surgical resection of the tumor combined with external radiation therapy and, in certain cases, chemotherapy. These treatments induce side effects and sequelae in the orofacial sphere, considerably altering the patient's quality of life. Mandibular ORN has been described since radiotherapy was shown to be an effective treatment for UAT cancers.

In recent years, the incidence rate of mandibular ORN has greatly reduced thanks to more accurate ballistics which spares more healthy tissue. The estimated incidence of ORN varies across institutions and time (reviewed in^1^). Unfortunately, a retrospective study has demonstrated that new radiotherapy techniques, such as Intensity-Modulated RadioTherapy (IMRT) or Volumetric Modulated Arc Therapy (VMAT), do not further reduce the incidence of mandibular ORN^1^. Mandibular ORN is one of the most feared sequelae of deleterious effects on bone metabolism and vascularization and causes extensive and irreversible bone necrosis. Patients who suffer from this disease present serious esthetic and functional defects, which can be life threatening.

Micro-anastomosed free-flaps and autologous bone grafts are standard reconstruction procedures. However, they cannot be performed on all patients due to the high risk of morbidity. Some encouraging results have been obtained with the PENTOCLO combined treatment^2^ but randomized clinical trials are still lacking, and this treatment may not be effective at advanced stages of the disease. There is therefore a real need to find new and effective treatments for mandibular ORN. Bone tissue is slowly but continuously remodeled and maintains its structural integrity by regulating the activity of osteoblast and osteoclast cells. Bone is also strongly interconnected with the vascular and hematopoietic systems. These different cell types have very different sensitivities to ionizing radiation, causing variable behaviors from one cell type to another that also vary over time. Indeed, osteoclast precursors and endothelial cells are described as radiosensitive cells compared to differentiated cells like osteocytes. However, the mesenchymal stromal cells (osteoblast precursors) are relatively radioresistant^3^ but irradiation induces modification of their differentiation capacities shifting differentiation toward adipocytes rather than osteoblasts^4^. That said, the integrated response of various cell compartments to tissue irradiation is not completely elucidated but evolves toward bone necrosis. Vascularization is involved in bone remodeling, transporting nutrients, growth factors and oxygen. Moreover, endothelial cells interact with and stimulate bone progenitors. Given the importance of vessels in bone physiology, Bras et al. hypothesized, as far back as 1990, that the alteration of the inferior alveolar artery and resulting tissue ischemia would cause ORN to progress on mandibular bone^5^. Improving our knowledge at cell and molecular levels would help us understand how the pathology evolves and highlight new therapeutic pathways. Moreover, with significant advances in biomaterial engineering, biotherapy and cell therapy, a real hope exists to cure the disease by combining various products. Thus, it is primordial to set up a pertinent animal model reproducing the human disease and allowing for the analysis of quantitative methods to test the efficacy of different combinations of treatments over time, based on different parameters.

The reference methods used to assess bone and vascular microstructure include 2-dimensional (D) histological sections, micro-computed tomography (µCT) imaging with or without contrast agents such as barium sulphate, enabling the visualization and quantification of blood vessels. Histomorphology has been considered as the gold standard for identifying and visualizing the microstructure of bone and blood vessels, in particular for small animals. However, this method can be subjective, thus it is frequently used qualitatively. Moreover, it cannot be used to assess bone changes over time unless many animals are euthanized.

In recent years, the development and the use of high resolution µCT imaging has increased considerably and is especially appropriate for bone assessment^6^. This 3D imaging technique is also a powerful tool for 3D vessel network analysis following the injection of a contrast agent. Moreover, with the improved sensitivity of µCT, the latest generation of µCT allows for both in vivo longitudinal studies and the analysis of ex vivo samples that can then be used for histology. µCT image analysis is now an essential asset for tracking in vivo longitudinal changes in bone mass and bone microstructure due to disease and/or bone regeneration processes, while reducing the use of laboratory animals. Today there is still a lack of in vivo methods that enable bone defects to be quantified at an early stage followed by the progression of mandibular ORN in animal models. Moreover, image analysis methods must be precisely defined according to the tissue (bone, vessels), the type of damage (weak, severe and extended) and the period of analysis.

In this study, we used the Perkin Elmer Quantum imager to assess mandibular bone damage in Lewis rats receiving 20 Gy, 25 Gy, 30 Gy and 40 Gy of irradiation with incident X-rays on the left mandible. Longitudinal analyses were performed at 3 Weeks (W), 8 W, 12 W and 16 W. At the final point, barium sulphate was injected intravenously for vascular network quantification.

Then, in vivo and ex vivo image analyses were compared to define the best ratio method for quantifying bone tissue damage. The benefit brought by the sub-reconstructions of volumes will be highlighted. The selected parameters are the robustness/strength of the scientific results, non-invasive approaches for animals and time savings for researchers. We also appraised progress on mandibular ORN based on clinical follow-up in animals.

## Materials and methods

### Animals

43 Adult Lewis rats from Janvier Labs (Le Genest-Saint-Isle, France) were used for all experiments. Animals were housed at the IRSN (Institute for Radiological Protection and Nuclear Safety) animal facilities accredited by the French Ministry of Agriculture for performing experiments on rodents. Animal experiments were performed in compliance with relevant guidelines and regulations and conformed with the ARRIVE guidelines and the French and European regulations on the protection of animals used for scientific purposes (EC Directive 2010/63/EU and French Decree 2013–118). All experiments were approved by the Ethics Committee of the IRSN #81 (approval number C92-032-01) and authorized by the French Ministry for Research under the reference APAFIS##6239-2016072812553546 v2.

Rats were housed in two-story ventilated cages (4 animals per cage). Animals were monitored (behavior, food intake) and weighed twice a week throughout the experiment. To facilitate rat feeding, kibbles were placed directly in the cage plus 30 g/per rats of high energy soft food (Transwean (M) IRR 829035, SDS, France) from the day of irradiation.

### Irradiation procedure

35 rats were irradiated on a medical linear accelerator (Elekta synergy) delivering 4MVp X-rays (mean photon energy about 1.3 MeV). Reference dosimetry measurements were taken using a 0.125 cc cylindrical ionization chamber calibrated in dose to water in a rat tissue-equivalent phantom placed on a plexiglass support. Measurements were taken 1 m from the source with a 2 × 3 cm irradiation field with an incident beam on the left mandibles. A dose rate of about 2.5 Gy/min in dose to water was used and rats were irradiated at 20, 25, 30, 40 Gy under an isoflurane inhalation anesthetic. Uncertainty for the dose rate measurement is about 5% at k = 2.

### Dental procedures

Three weeks after irradiation, the second left median molar of all rats was extracted under general anesthetic (75 mg/kg Ketamine and 0.5 mg/kg medetomidine by intramuscular injection) followed by reversion (with 1 mg/kg atipamezole, subcutaneous). Buprenorphine (0.05 mg/kg, subcutaneous) was injected before extracting the molars. As rats have difficulty feeding with hard food (kibbles), their upper incisors do not wear out, they continue to grow, until they hurt their mandibular skin. Thus, they were cut with a diamond bur (NE116, NKS-Nakanishi, France), every 3 weeks under an isoflurane general anesthetic after a prior injection of buprenorphine. While under anesthesia, cutaneous damage was observed and managed with vetedine.

### Contrast agent injection, euthanasia and mandible preparation

Under general anesthesia with isoflurane and after the subcutaneous administration of 0.05 mg/kg buprenorphine, intracardiac perfusions were performed with barium sulphate as described in Blery et al.^7^. At the end of the procedure, mandibles were harvested, pictures of the explanted mandibles were taken using binocular imaging (Stemi SV11 Apo, Zeiss, Oberkochen, Germany). Mandibles were fixed in a solution of 4% formaldehyde over 7 days, then treated with papain for 16 h to remove as much flesh as possible and finally stored in 70% ethanol. For µCT acquisitions, as much soft tissue as possible was carefully removed and the left and right mandibles were separated.

### Micro-CT imaging

An In vivo micro-scanner platform was used for a longitudinal study. At 3, 8, 12 and 16 weeks (W), rats were anesthetized under isoflurane (2%; 1 l/min O_2_) throughout scanning. At 3 weeks, scans were performed before dental extraction. Follow-up µCT images of the group irradiated at 40 Gy were recorded at the “Imagerie du Vivant, Université Paris Descartes, Faculté de Chirurgie Dentaire” dental imaging platform using a QuantumFX micro-CT scanner (Perkin Elmer) under isoflurane anesthesia. Images were acquired at 90 kV, 160 mA, with no additional filtration, for 2 min. Views are reconstructed in a stack of images containing 512 × 512 × 512 voxels of 80 µm. Images were acquired 8, 12 and 16 weeks after irradiation. For in vivo µCt images of the other three groups (20, 25 and 30 Gy) and for ex vivo µCT images, acquisitions were recorded using the Quantum GX2 (Perkin Elmer). Images were acquired at 90 kV, 88 µA, with an additional filtration of 0.06 mm of Cu and 0.5 mm of Al. For the follow-up, µCT images of the groups irradiated at 20, 25 and 30 Gy were recorded under isoflurane anesthesia with a FOV (Field of View) of 72 mm, a reconstruction of 45 mm, for 2 min. Views are reconstructed in a stack of images containing 512 × 512 × 428 voxels of 90 µm. For ex vivo images, all images were acquired with a FOV of 36 mm, a reconstruction of 36 mm over an acquisition time of 57 min. Views were reconstructed in a stack of images containing 512 × 512 × 428 voxels of 72 µm. In order to characterize the bone and vascular defects specifically in the ORN development region, sub-volume reconstructions were performed. This region includes the 3 molars area. For that, to increase the voxel size of the existing ex vivo images and improve the detection, visualization and quantification of the vascular system on the bone, smaller region of interest (ROI) on the ex vivo CT scan were selected for sub-volume reconstruction, enhancing the image quality by reducing voxel size from 72 to 25 µm.

### Images analysis

Images were analyzed using AnalyzePro version 1.0 and Analyze12 version 12.0 software (AnalyzeDirect; https://analyzedirect.com) for 3D analysis (volume quantification) and the 3Dslicer version 4.10.1 (https://www.slicer.org) software for 2D measurements (distance).

2D measurements were taken to quantify local bone loss. The images were reformatted for this purpose using 3Dslicer software in order to always take the measurements in the same cutting plane. Then, the local bone loss was quantified by measuring the distance between the teeth and the bone under the first molar. All measurements were performed by the same operator.

For 3D µCT image analysis, we quantified the bone volume on in vivo images and the bone and vessel volumes on ex vivo images.

When quantifying bone volume on the in vivo images, the semi-automatic software option was used to segment each mandible as an object, always using the same threshold range for all rats for comparison purposes. Then, we manually excluded the incisors by contouring, on the frontal section, 1 slice in 4, and by propagating the volume following this section. The resulting total bone volume on in vivo images factors in bone and molars.

On ex vivo images, the same method was used to extract the total bone volume and the incisor. Thanks to better image quality and smaller voxel size, the molars were also excluded manually. Then, to extract the vessel volume, we first used the semi-automatic software option allowing for 3D spatial connectivity to link together voxels having gray levels in the same range. Even if the main part of the vascular network was segmented, as the vascular volume was not continuous especially for irradiated rats, the rest of the vascular network that had not been considered was segmented manually. To make sure that the entire vascular network was correctly segmented, each slice was checked one by one.

### Histology

After µCT image acquisition, mandibles were decalcified in EDTA (MoL-DECALCIFIER 51413, Milestone, BG) for 21 days and then paraffin embedded, and transversal sections were taken from basal to alveolar bone. Transversal 5 µm mandible sections were stained with HES (Hematoxylin–Eosin-Saffron). Representative pictures were taken with NanoZoomer S60 and NDP viewer1 (Hamamatsu, France).

### Statistical analysis

Data are given as means ± SEM. When the group samples succeed in the Shapiro/Kolmogorov normality test, statistical analysis was performed by ordinary one-way ANOVA (Tukey’s multiple comparison) with a level of significance of p < 0.05. For the longitudinal study, the sham group, did not pass the normality test. In this case, the non-parametric Kruskall-Wallis test has been used. Concerning the weight curves according time, Friedman non-parametric test analysis have been used.

## Results

### Clinical aspects of mandibular ORN in rats

**Figure 1:**
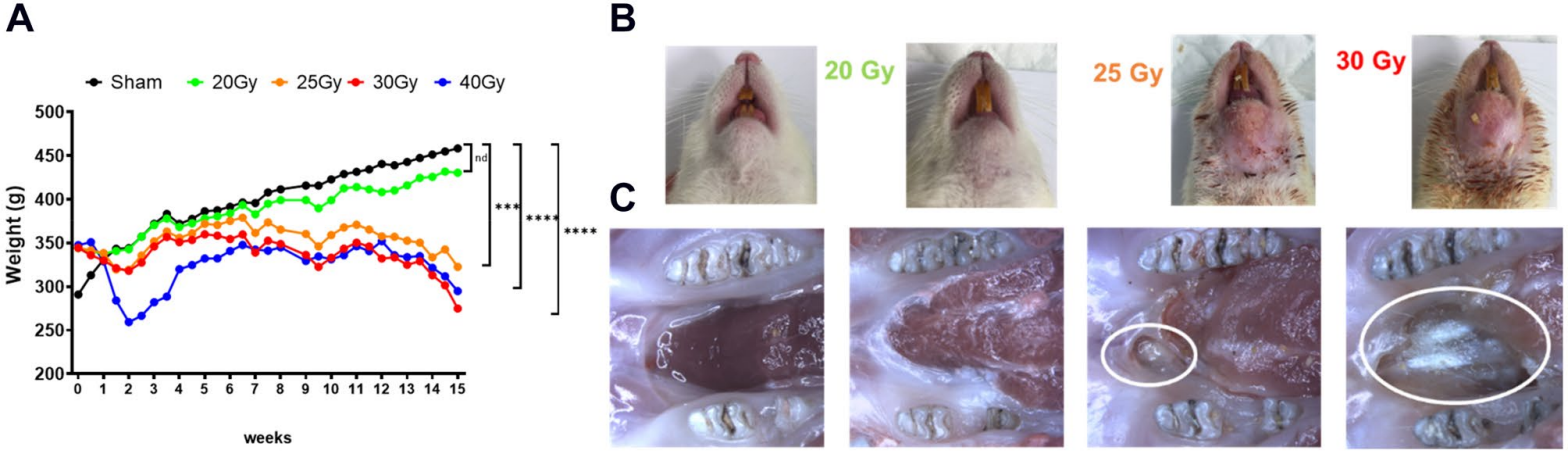
Clinical follow-up of animals according to irradiation dose. (**A**) Animal weight curves. Statistical analysis: Friedman non-parametric test. (**B**) Representative pictures of external cutaneous damage. (**C**) Representative pictures of buccal damage after explantation. Mucosal ulcers and bone exposure are highlighted by white circles.

Rats irradiated at different doses were closely monitored over 16 weeks (W). In particular, they were weighed twice a week (Fig. 1A). We rapidly observed, following irradiation, a decrease in the animal’s weight correlated with an increase in the irradiation dose. After 3 weeks, rats regained weight whatever the irradiation dose, up to 8 weeks. Then, we observed discrepancies between 20 Gy irradiated rats (whose curve resembles the sham rats) and 25, 30 and 40 Gy irradiated animal weight curves. It appeared that 40 Gy irradiated rats lost a significant amount of weight in the weeks following irradiation compared with 25 and 30 Gy irradiated rats. Moreover, despite all supportive care, 40 Gy irradiated animals were in bad general condition compared to the other groups (data not shown).

Cutaneous lesions were also observed as reported in Fig. 1B. The first observable effect was that irradiated rats lose their hair located within the irradiation window and we could observe hair regrowth for 20 Gy contrary to 25, 30 and 40 Gy irradiation doses. We noticed that irradiated rats suffer from trismus and the mandibular incisor stops growing. As in humans, rats have difficulty feeding. From 25 Gy dose, dry then moist desquamations were observed, with deeper lesions as the irradiation dose increased (Fig. 1B). We also observed orostoma (communication between the oral cavity and the skin of the neck) solely for 40 Gy (data not shown). After explantation, at the 20 Gy dose, no mucosal lesion was clinically observed during buccal examination (Fig. 1C). However, at a dose of 25 Gy, 66.6% of animals exhibit mucosal ulcers. From 30 Gy, all rats suffer from severe mucosal lesions. Thus, clinical observations highlight discrepancies between a 20 Gy dose that does not induce mandibular ORN and the other irradiation doses.

### Histology of mandibular bone according to the different irradiation doses

**Figure 2:**
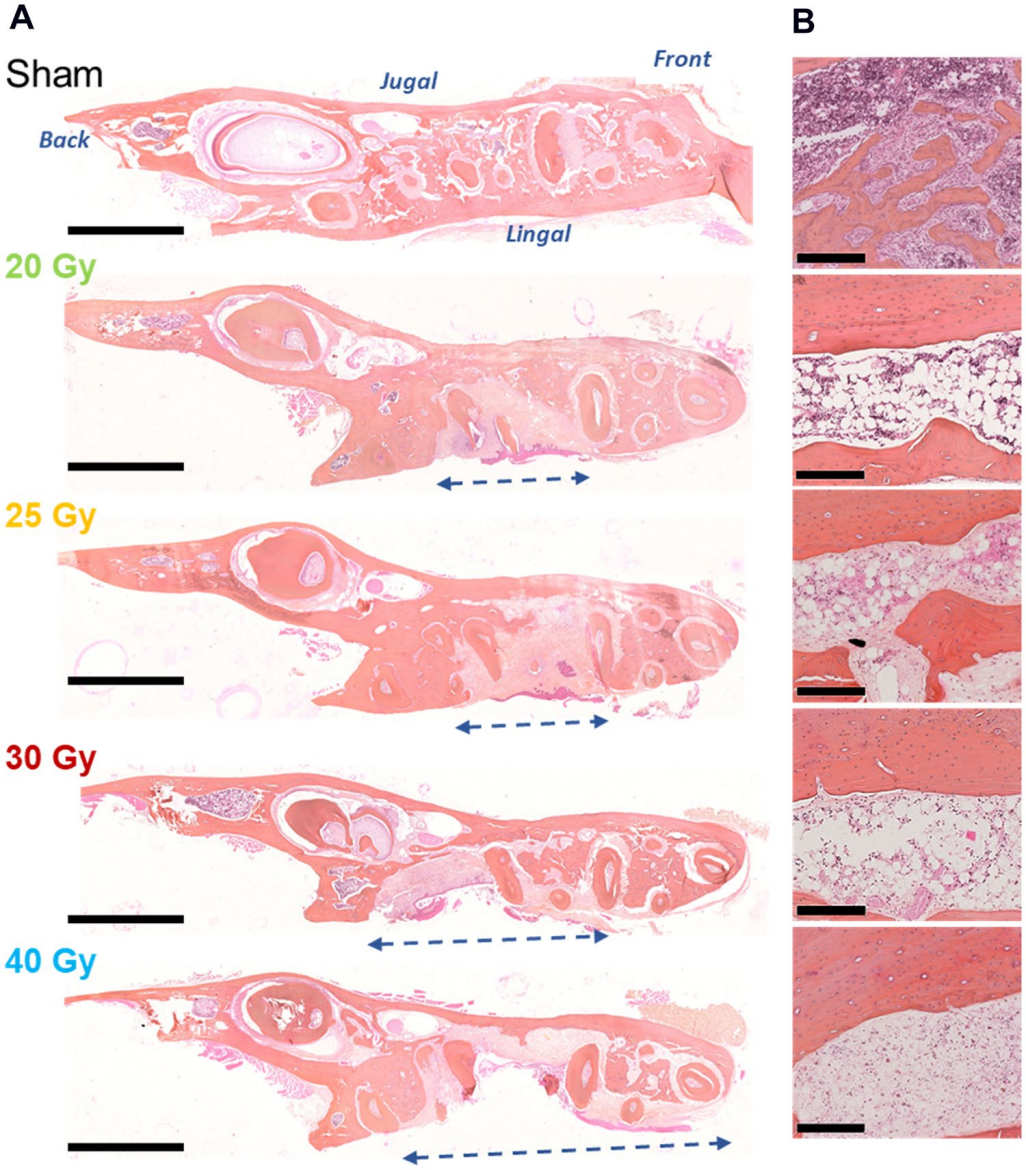
Histological analyses on transversal section of left mandibles. Representative HES staining in Sham and irradiated animals after 16 W. (**A**) of upper transversal series of the left mandible at different irradiation doses (alveolar side). Blue arrows show bone damage expansion for each irradiation dose. Scale bars represent 2.5 mm. (**B**) Bone and bone marrow composition (basal side). Scale bars represent 250 µm.

The mandibles were analyzed by histology after 16 W for each irradiation dose. After irradiation, for the left mandibles, we observed bone loss at the location of the molar extraction. The extent of bone loss is greater the higher the radiation dose (Fig. 2A). Bone is replaced by fibrous tissue, damage is more pronounced on the lingual side and the upper transversal side (alveolar bone) of the mandible.

In sham mandibles we observed trabecular bone with numerous cavities, whereas in irradiated mandibles, fewer cavities containing bone marrow were observed (Fig. 2B). In irradiated bone, we observed empty osteocyte lacunae. Bone marrow located in the basal side was also altered according to the irradiation dose. At 20 Gy, more adipocytes were observed in the bone marrow compared to the sham. At 25 Gy, fewer cells were found in the bone marrow, at 30 Gy in addition to all this the bone marrow was hemorrhagic, rare hematopoietic cells persisted and at 40 Gy the cavity was mainly composed of fibrous tissue. Bone loss was also observed on right mandibles to a lesser extent (Fig. S1) and a thinning of the mandible was observed from a 30 Gy irradiation dose.

### Detection of bone defects according to variation in irradiation doses according to CT image definition

**Figure 3:**
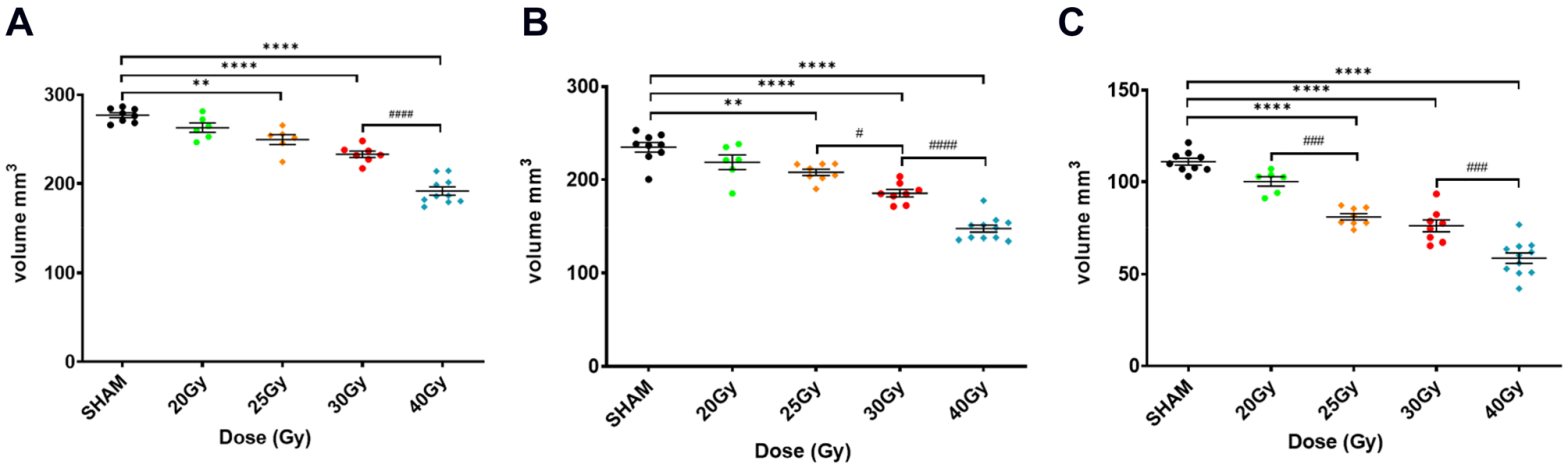
Bone volume quantification according to doses 16 W after irradiation using three CT images. (**A**) Quantification of total mandibular bone volume according to in vivo CT images acquired with a voxel size of 90 µm and (**B**) ex vivo total CT images acquired with a voxel size of 72 µm. (**C**) Bone volume quantification focus on region of interest (ROI) design on ex vivo CT scans (reconstructed voxel size: 25 µm). N = 8 animals for Sham, 25 Gy and 30 Gy; N = 6 animals for 20 Gy; N = 10 animals for 40 Gy. * versus sham; # versus groups. Statistical analysis: Normality tests Shapiro /Kolmogorov then One-way ANOVA.

In order to evaluate the detection threshold for bone defects according to the irradiation dose, we analyzed three types of CT images: (i) in vivo CT images with a voxel size of 90 µm, (ii) ex vivo CT images with a voxel size of 72 µm and (iii) ex vivo CT images with a voxel size of 25 µm obtained after reconstructing the ORN development region, i.e. in the extraction area of the second molar. To be able to compare the bone volume damaged, analyses were performed at the final endpoint, 16 W after irradiation. As shown in Fig. 3 and as expected, we observed a decrease in bone volume as the irradiation dose increases. This result was obtained for each type of CT image analyzed; however, interesting differences can be noticed.

- The in vivo CT scan images at 90 µm were acquired in only 2 min to scan the total heads of the rats (Fig. 3A). Using this method, for the 20 Gy irradiation dose, we observed non statistical bone loss compared to sham rats. For 25 Gy, 30 Gy and 40 Gy, statistical decrease with sham rats was detected with 9.9% (p = 0.0017), 15.86% (p < 0.0001) and 30.83% (p < 0.0001) of bone loss respectively (Fig. S2A). This method detects significant bone volume differences between 30 and 40 Gy (p < 0.0001).
- Ex vivo total mandibular CT scans were acquired with a voxel size of 72 µm with an acquisition time of 57 min. This improved the quality of the images, the signal-to-noise ratio and provided a more accurate segmentation (Fig. 3B). This method detected differences between sham and irradiated groups in the same way as the previous one. The bone loss compared to sham are for 20 Gy: 6.91% (ns), 25 Gy = 11.49% (p < 0.022), 30 Gy = 21.04% (p < 0.0001), 40 Gy = 37.27% (p < 0.0001) (Fig. S2B). However, differences between two doses were detected for lower bone loss than previously. Indeed, this method statistically identifies differences between 25 and 30 Gy (p < 0.0183).
- ROI design on ex vivo CT scans were performed on the previously acquired images and focused on the ORN development region. The voxel size achieved with these sub-reconstructions is about 25 µm (Fig. 3C). At 20 Gy, non-significant bone volume loss of 9.7% compared to the sham group was detected (Fig. S2C). From the 25 Gy dose, the differences between the sham group and irradiated groups were statistically significant with (p < 0.0001). In the same way, high statistical differences (p = 0.0003) were detected between 20 and 25 Gy irradiation doses.

To conclude, 16 W after irradiation, the three methods detected statistical bone damage from a dose of 25 Gy. The use of ROI on ex vivo CT scan is the only method that detects differences for the lowest radiation doses (i.e. between 20 and 25 Gy).

### Detecting vessel defects according to variation in irradiation dose

**Figure 4:**
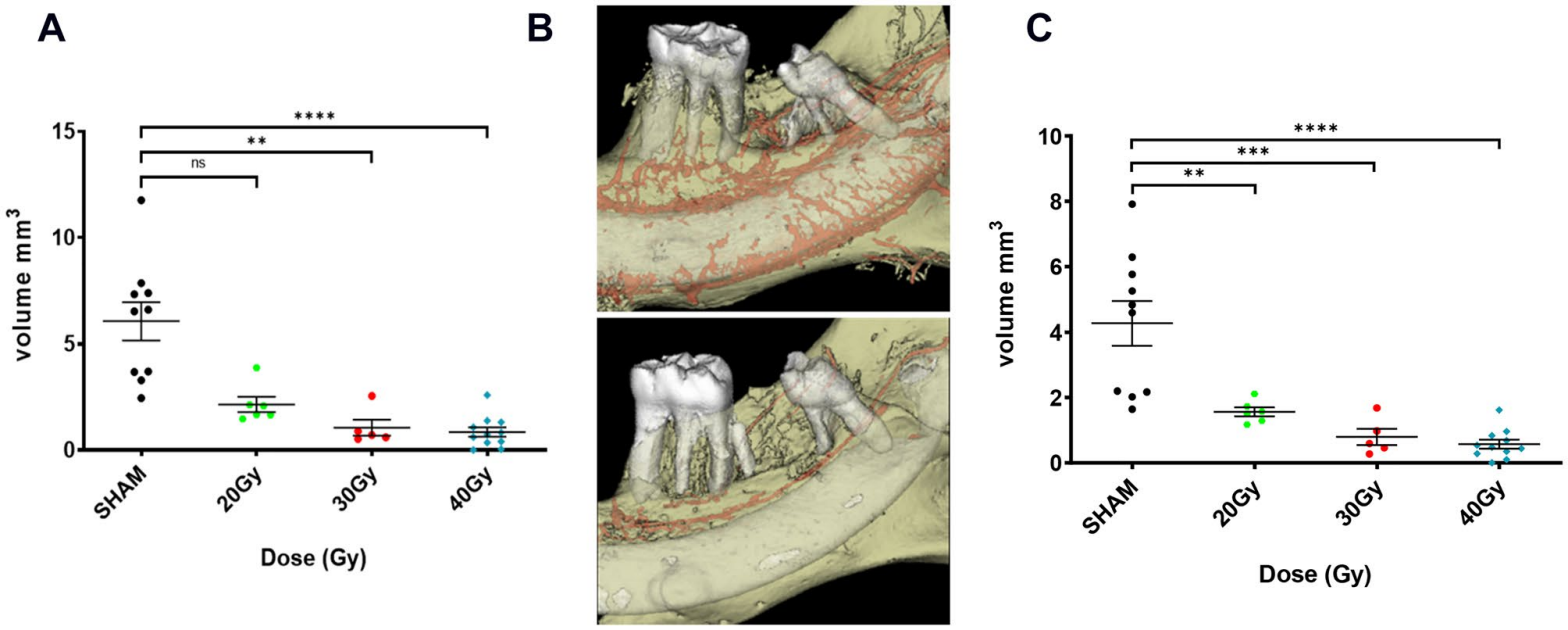
Volume quantification of the vessel network according to doses 16 W after irradiation. (**A**) Quantification of total mandibular vessel volume according to ex vivo CT images acquired with a voxel size of 72 µm. Statistical analysis: Kruskall Wallis non-parametric test. (**B**) Representative visualization of the vessel network (red) centered on ROI on ex vivo CT scans (72 µm) in sham (up) and irradiated 40 Gy (down). (**C**) Vessel volume quantification focused on ROI design on ex vivo total CT images acquired with a voxel size of 72 µm. Statistical analysis: Shapiro normality-test then One-way ANOVA. N = 10 animals for Sham, N = 6 animals for 20 Gy; N = 5 animals for 30 Gy and N = 11 animals for 40 Gy. * versus sham.

As vessel detection requires barium sulphate intra-cardiac perfusion, only ex vivo CT scans were performed. We compared ex vivo images acquired for the total volume of the mandible with images centered on the molar region after sub-reconstructing volumes (72 µm for total–25 µm for ROI). We detected a drastic effect of irradiation from a 20 Gy irradiation dose using these two analysis methods. We observed that the difference is not significant at the 20 Gy dose using the total vascular volume quantification (Fig. 4A–C).

We quantified, respectively for 20 Gy, 64.5% (total vessel volume) and 63.4% (volume in ROI) of vessel volume loss. For 30 Gy, 82.6% and 81.3% and for 40 Gy, 86% and 86.6% were quantified. Although the two methods used for segmentation enable us to quantify loss of vascular network volume at the same extent, the analyses performed on ROI allowed us to more accurately segment and seems to suggest a radiation dose effect between 20 and 30 Gy on the volume of vessels.

### Determination of the initiation of bone defects and progression using in vivo CT scan images

**Figure 5:**
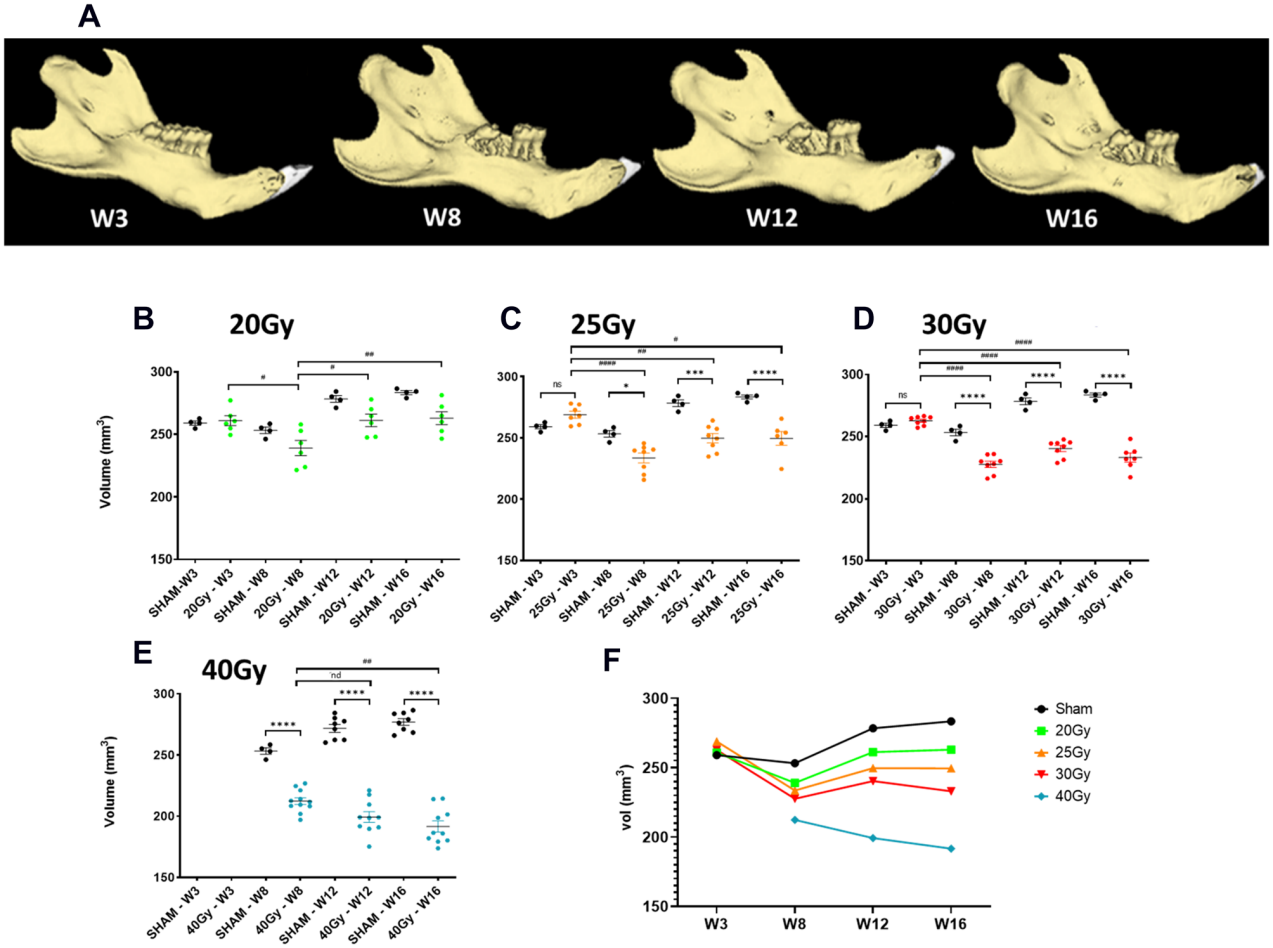
In vivo longitudinal study of total mandibular bone volume from 3 to 16 W. (**A**) Representative 3D reconstituted pictures of left mandible of 25 Gy irradiated rats among time. Graphics representing total bone volume of left mandible for (**B**) 20 Gy; N = 6 animals (**C**) 25 Gy; N = 7 animals (**D**) 30 Gy; N = 8 animals (**E**) 40 Gy; N = 10 animals compare to Sham N = 4. Statistical analysis: Shapiro normality test then One-way anova. (**F**) Representative graphic of total mandible change in bone volume over time including all irradiation doses. * versus sham; # versus groups. nd: no difference.

**Figure 6:**
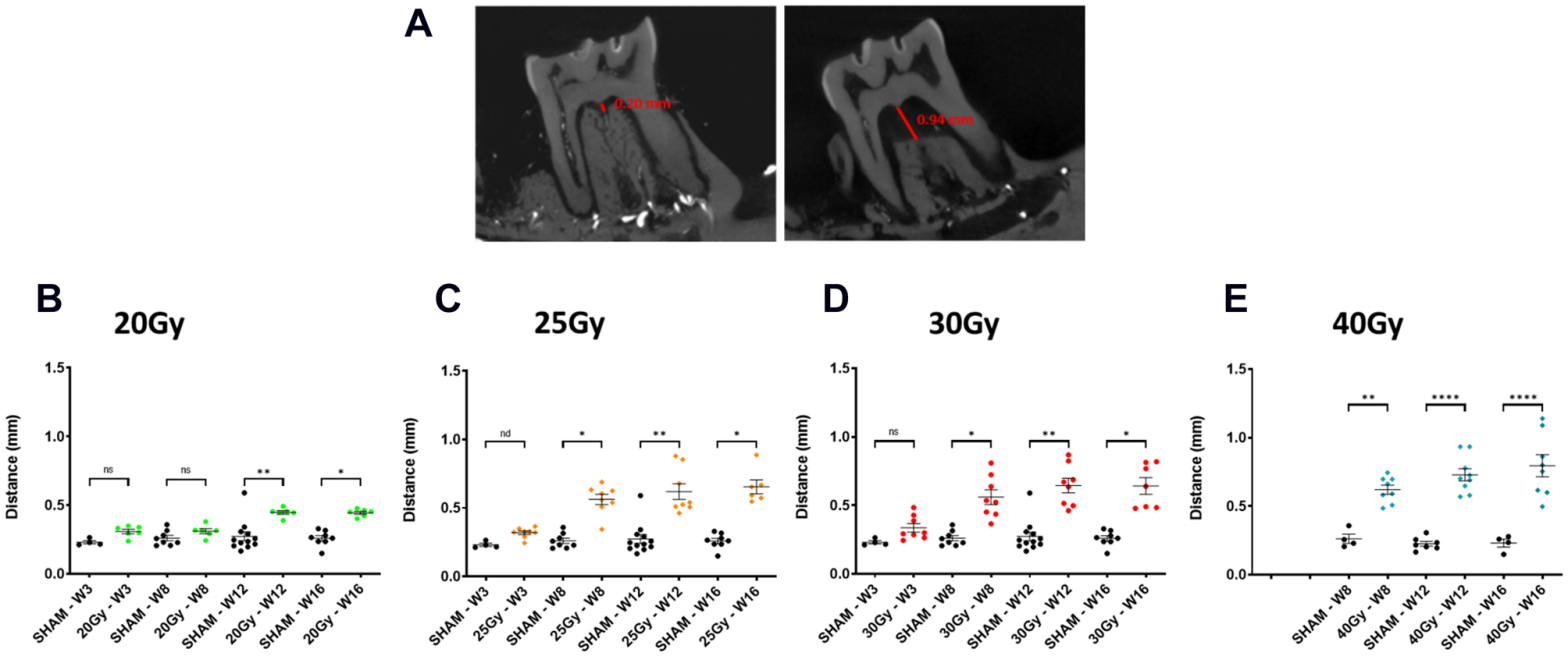
In vivo longitudinal study of distance measurement on 2D images from 3 to 16 W. (**A**) Representative pictures showing the distance measurement between the first molar and the underlying bone in sham (left) and irradiated animals (right). (**B**) Graphic representing localized bone loss for 20 Gy; N = 6 animals (**C**) 25 Gy; N = 7 animals (**D**) 30 Gy; N = 8 animals (**E**) 40 Gy; N = 8 animals compare to Sham N = 4. Statistical analysis: Kruskall Wallis non-parametric test * versus sham.

To detect the initiation and progression of mandibular ORN in animals, we took advantage of the in vivo CT scan to study bone changes over time. We compared the whole volume of the mandible (Fig. 5—3D analysis) and the distance under the first molar between tooth and bone to assess local bone loss (Fig. 6—2D analysis) at different end points; 3 W (except for the 40 Gy group), 8 W, 12 W and 16 W.

Regarding the measurement of the entire volume of the mandibular bone (Fig. 5), the sham group showed no change between 3 and 8 W due to molar extraction 3 W after the scan. Indeed, the absence of modification is the concomitant action of the increase in bone volume due to animal growth and a decrease in the signal due to molar extraction. Then, at 12 W and 16 W, as expected, bone volume increased compared to 8 W (p = 0.0289 and p = 0.0047 respectively), in the same way as animal growth. In accordance with our previous study, for all the irradiated groups, whatever the irradiation dose, we observed a decrease in bone volume at 8 W compared to 3 W, due to molar extraction and irradiation^8^.

After 20 Gy irradiation, we did not detect any significant difference with regard to the sham group regardless of the time period analyzed (Fig. 5A). This demonstrates that, for a 20 Gy dose, bones heal after tooth extraction as in the sham animals. For a 25 Gy dose, no significant differences were observed at 3 W compared to the sham group, however significant bone defects were observed at 8 W with p < 0.0262 then significance with p < 0.0003 at 12 W and p < 0.0001 at 16 W compared to the sham group (Fig. 5B). The increase in difference compared to the sham group over time demonstrates a worsening of bone loss. In particular, curve variation analysis of 25 Gy irradiated rats showed no difference over time from 8 W, demonstrating the absence of bone healing after 25 Gy irradiation and tooth extraction. For 30 Gy and 40 Gy highly significant bone defects were detected as early as 8 W. For 40 Gy, we noticed a strong reduction in the bone volume at 12 W and 16 W compared to 8 W, highlighting bone degeneration (Fig. 5C,D). To conclude, overall analysis of the curves clearly demonstrates that at 20 Gy, bone can regenerate following molar extraction and irradiation. 25 Gy and 30 Gy irradiation doses are intermediate with no major change in bone volume from 8 W and at 40 Gy the mandibular bone substantially degenerates over time (Fig. **5**F). These results were compared against the 2D measurements of the distance between the first molar and the underlying bone (Fig. 6). This method specifically targets bone resorption near the extracted zone.

For the sham group, the distance measured is short and generally constant over time. For all irradiated groups, we detected a significant increase in the distance compared to sham from 12 W (Fig. 6B–E). Interestingly, this 2D measurement is the only one that highlights bone resorption at the 20 Gy dose from 12 W after irradiation. Thus, 2D distance measurement seems appropriate for quantifying local and weak bone lesions. However, maximal distance is rapidly reached limiting the quantification of significant bone damage.

### Differential bone defect over time on left versus right mandible

Rats were irradiated with an incident beam on the left mandible that crosses out through the right mandible, leading to irradiation of both mandibles. First, we analyzed the impact of irradiation for the right mandible. 3D volume analyses detected a statistical bone defect on the right mandible for 25 Gy only at the latest time point (16 W) (Table 1A). We observed that 2D distance measurement on the right mandible reflects statistical differences with the sham group for 20 Gy and 25 Gy at 12 W and for 30 and 40 Gy statistical differences with the sham group are detected as 8 W (Table 1B).

**Table 1:** Tables summarizing statistical differences in bone damage on the right (RM) and left mandibles (LM).

	W3	W8	W12	W16
**(A) Total volume between SHAM and RM**				
20 Gy	nd	nd	*	**
25 Gy	nd	nd	nd	*
30 Gy	nd	***	****	****
40 Gy	No data	No data	****	****
**(B) Distance between SHAM and RM**				
20 Gy	nd	nd	****	****
25 Gy	nd	nd	**	*
30 Gy	nd	*	****	****
40 Gy	No data	*	*	**
**(C) Total volume between RM and LM**				
20 Gy	nd	nd	nd	nd
25 Gy	nd	*	**	**
30 Gy	nd	****	****	****
40 Gy	No data	****	****	***

Differences on right mandible between Sham and irradiated animals according to irradiation doses over time (A) for total bone volume and (B) for distance measurements. nd (no difference) or * versus sham. (C) Table summarizing statistical differences in total bone volume as analyzed between the left and right mandibles in irradiated animals at different doses and over time. nd (no difference) or * left versus right. Statistical analysis: Normality tests Shapiro /Kolmogorov then One-way ANOVA for all tests except for comparing the distance parameter on the right mandible between the Sham and 25 Gy irradiated rats, we used the Kruskall Wallis non-parametric test.

These results confirm the high sensitivity of 2D distance measurements for minor injuries. Our irradiation model induces bone defects on the right mandible even if no teeth are extracted. However, total bone damage is less pronounced on the right mandible than the left mandible (Table 1C). Total bone volume measurements demonstrated statistical differences between the left and right mandibles from 25 Gy irradiation dose and after 8 W of irradiation.

## Discussion

ORN is a particularly morbid late effect of radiation therapy for UAT cancers that seriously impacts functional and esthetic outcomes for patients. ORN is a progressive disease leading to bone necrosis in an irradiated field unrelated to tumor recurrence and that fails to heal over 3 to 6 months. While clinical definition is almost consensual, several theories have been proposed regarding the physiopathology of ORN^9^. The lack of knowledge regarding mechanism-based progression of the disease leads to inadequate pathology staging systems and inappropriate adaptation of patient management. Moreover, there is a real clinical need for a pertinent animal model in order to develop new treatments based on advances on biomaterials, biomolecules and cell therapy. In this study, we refined a mandibular ORN rat model using different irradiation doses and performed the first in vivo longitudinal study from 3 weeks to 4 months.

Many ORN rat animal models have been proposed in previous studies, including our study, using different irradiation doses, fractionation, beam energies, trauma and strain of rats^10–16^. Today, no consensus exists regarding the mandibular ORN model, and most of these studies demonstrate bone defects characterized by histology or µ-CT in endpoint analysis (in general, 8 W after irradiation). However, primordial discrepancies could be observed between these models and their designation as ORN models could be questioned. Indeed, 20 Gy mandibular irradiation and extraction of the three molars induced bone volume differences between groups^11^. However, as no difference was observed compared to the sham group, the part due to irradiation could be negligible with regard to the large teeth extraction procedure. Studies using 30 Gy or higher irradiation doses demonstrated large bony defects, bone exposure was often observed but induced high mortality rates (up to 43% mortality^10^). In the current study, we analyzed dose ranges from 20 to 40 Gy, delivered with a medical accelerator (4MVp X-rays) to the left mandible associated with one median molar extraction to define the minimal irradiation dose that induces ORN, as defined in humans. In view of testing new treatments and particularly cell transplantation, we used inbred Lewis rats. A previous study developed an athymic ORN rat model, to avoid cell or biomaterial rejection^15^. However, as the immune system is a powerful inducer of bone repair, the use of immunocompetent animals seems appropriate for mimicking the different events that occur in humans. In our study, regardless of irradiation dose, no animal death was observed for up to 15 W. Then, we observed 25%, 37.5% and 18% mortality for 25, 30 and 40 Gy groups. With a view to refining the study in terms of animal ethics, future studies will be terminated before 12 W. In our in vivo µ-CT longitudinal study, associated with clinical examination, we defined 25 Gy as the first dose inducing ORN in rats as described in clinical practice. Localized bone defects can be detected with 20 Gy dose using the 2D distance measurement; however, bone regenerative process takes place contrary to the ORN definition (Fig. 5A).

As in numerous studies, our model used an external beam entering on the left mandible and passing through to the right mandible. We also focused on evaluating bone volume for the right mandible. We demonstrated that bone defects appear at the same time on the right mandible, however this damage is statistically less significant than on the left mandible. This result is in agreement with the latest definition of mandibular ORN that does not consider bone exposure in the definition of the disease^17^. Indeed, bone architecture modifications and osteopenia have been reported in some patients treated by radiotherapy for cancer, including pelvic and breast cancer, demonstrating the direct impact of ionizing radiation on bone physiology^18^.

In this study, we compared in vivo (90 µm) to ex vivo (72 and 25 µm) CT scans to define the trade-off between accurate bone defect quantification and the in vivo longitudinal study. Indeed, numerous methods of image analysis can be used. They must be precisely defined according the tissue, the type of damage and the time of analysis. µ-CT image analysis is quite challenging, and few reports detail the description of the methodological steps used to analyze these images^19^. However, the use of standardized assessment and consistent parameters to analyze mandible bone defects using µ-CT imaging could help in comparing studies and the benefits of therapeutic approaches. The analysis methods used in this study detected significant bone volume decrease from 25 Gy irradiation. To improve the quantification of total mandibular bone volume, incisors were contoured and excluded. Indeed, irradiation leads to the opacification of incisors that counterbalances signal loss due to bone defects induced after irradiation. Incidentally, the longitudinal study demonstrated significant differences after 25 Gy irradiation as early as 8 W, whereas without incisor contouring, differences are only observed for the longer period (16 W) and thus greater damage (data not shown). The molars are smaller than incisors in rats and more difficult to contour accurately due to the quality and voxel size of the images. They were not excluded for quantification due to their minor influence on results and the time-consuming operation.

As expected, we observed that increasing image definition, in particular using 25 µm voxel size, improves the detection of differences between the irradiation doses, and significantly so for lower irradiation doses. Indeed, definition of ROI highlights statistical differences between 20 and 25 Gy at 16 W. In vivo CT scan image analysis can only be used to detect significant differences for higher doses of irradiation and the longest periods analyzed (Supplementary Fig. 3). It cannot detect differences between 20, 25 and 30 Gy doses whatever the time point analyzed (Fig. 5B–F and Supplementary Fig. 3). Thus, we completed our quantification method by defining a pertinent measurement on 2D in vivo CT scan images that quantified the distance between the first molar and the underlying bone. This measurement specifically targets the irradiated region and the bone lesion induced by tooth extraction. We concluded that this method is appropriate for detecting small-scale bone damage at lower doses or at early time points of the disease, as shown for 20 Gy on the left and right mandibles.

The vascular system is closely linked to bone physiology. Moreover, studies have demonstrated that ionizing radiation alters endothelial cells initiating organ damage^20,21^. However, data are lacking concerning flat bone tissue and this model could be useful in understanding the interplay between angiogenesis and osteogenic pathways in ORN progression. In this study, we showed a drastic effect of irradiation at 20 Gy on the mandibular vasculature after agent contrast injection and µ-CT scan image analyses. We and other authors have demonstrated that using barium sulphate as the contrast agent gives good results for vessel visualization in rats^7,8,22^. However, given the voxel size of the images at 72 µm, vessel segmentation was difficult, especially for irradiated rats where the vascular network was strongly impacted. This voxel size did not allow us to quantify the smallest vessels as they were not detectable on the CT images. In particular, we highlighted the fact that ROI design leading to a voxel size of 25 µm allows for more accurate detection and quantification of the vessel network. The results showed a drastic effect for irradiation from 20 Gy (16 W), however µCT and immunohistochemistry analyses at earlier time points will be necessary, in the future, to improve our knowledge of the impact of irradiated vessels on bone physiology. It would be interesting to find out if bone destruction is caused directly by irradiation injury to the cells involved in bone remodeling, indirectly due to irradiation-induced vascular injury or to a combination of effects.

In vivo longitudinal µCT study is a useful way to decrease animal use, since measurements are taken on the same individual, at the same location over time and also have the potential to differentiate bone changes pre-dating the treatment from those occurring after treatment onset, potentially detecting preventative versus restorative therapeutic effects. However, repeated CT-scans delivering radiation doses could alter bone microstructure. Several rodent studies have been performed to assess the impact of repeated µCT acquisitions on the whole body or the exposed limb^23–25^. Depending on the model (mouse/rat), the age, the number and time interval for CT-acquisitions and the CT-scan dose, different responses to radiation doses have been reported leading to undesirable side effects^24,25^ or not^23^. Even if a high radiation dose provides better images, especially for bone damage quantification, it is necessary to find a compromise between image quality, CT-scan dose and the number of acquisitions during the longitudinal study. In our study, we decided to perform a 2-min CT scan corresponding to a dose of 0.1 Gy in dose to water using Quantum GX2, to limit dose deposition, especially on bone structure, when using low X-ray energies (effective energy of 32.9 keV (Cu)). Indeed, at these energies, dose deposition is mainly done by photoelectric effect (Z^3^ dependence), so a small variation in the composition of bone tissue could lead to a large variation in the absorbed dose to the bone. Four CT scans were performed during the longitudinal study, leading to an additive dose of 0.4 Gy in dose to water using Quantum GX2. Experimental dose measurements and/or Monte Carlo simulations could be performed to know the exact dose for the bone structure. However, as the dose deposition is relatively low compare to our experimental irradiation dose, for future studies and to increase the quality of the in vivo images, an acquisition time of 4 min will be used.

In this study, we defined the minimal irradiation dose, 25 Gy located on the mandible delivered with a medical accelerator, inducing significant bone defects without regenerative capacities in inbred Lewis rats. We also defined the parameters for CT image analysis allowing in vivo longitudinal mandibular bone volume quantification with precision as close as possible to the ex vivo methods that require animal euthanasia. Hence, total mandible volume quantifications for 90 µm acquired images, after incisor contouring, quantify bone damage, rather at the later stage (8 W to 16 W). Thus, to complete this analysis and discriminate minor and early bone damage, using in vivo µCT images, we have developed 2D measurements that could be applied to the left and right mandibles. This refined pre-clinical rat model associated with accurate quantification methods will be very useful for quantifying new therapies or combinations of treatments to limit or cure mandibular ORN while limiting the use of laboratory animals.

## Supplementary Information

Supplementary Information.
