# Supplementary material for: Refining the mandibular osteoradionecrosis rat model by in vivo longitudinal µCT analysis
Source: Sci Rep. 2021 Nov 15;11:22241. doi: 10.1038/s41598-021-01229-y (PMC8594779; doi:10.1038/s41598-021-01229-y)
Supplement: Supplementary file 1 — Supplementary Information. [file 41598_2021_1229_MOESM1_ESM.docx]

**Supplementary data**

**Supplementary Figure 1: Representative pictures of bone damage induced after different irradiation doses on the right mandible.** HES immunostaining on transversal section of right mandible - Scale bar represent 2.5 mm

**
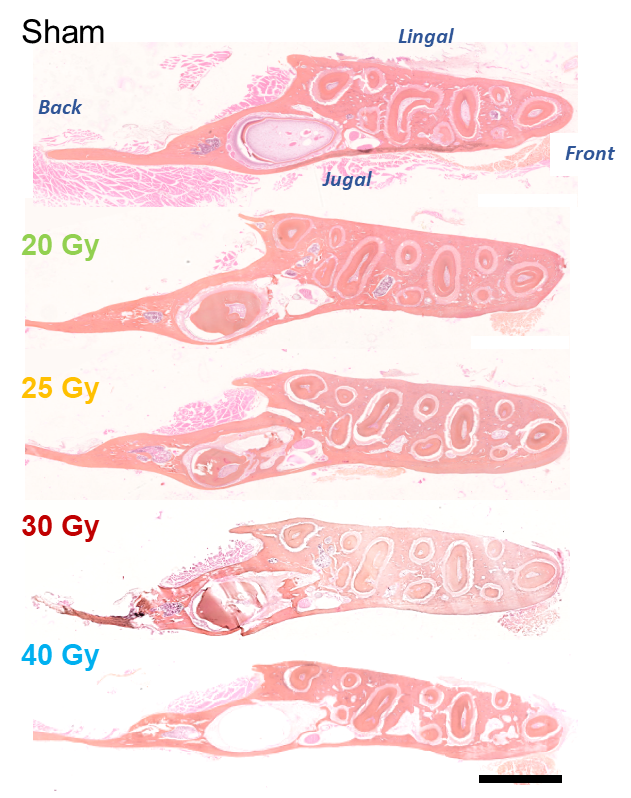
**

**Supplementary Figure 2: Graphics representing the percentage of volume bone loss compared to sham animals (left mandible).**

**(A)** *in vivo* CT images acquired at 90 µm resolution and **(B)** *ex vivo* total CT images acquired at 72 µm resolution. **(C)** quantification focus on ROI design (25 µm) on *ex vivo* CT scans. N= 8 animals for Sham, 25 Gy and 30 Gy; N= 6 animals for 20 Gy; N=10 animals for 40 Gy.

**
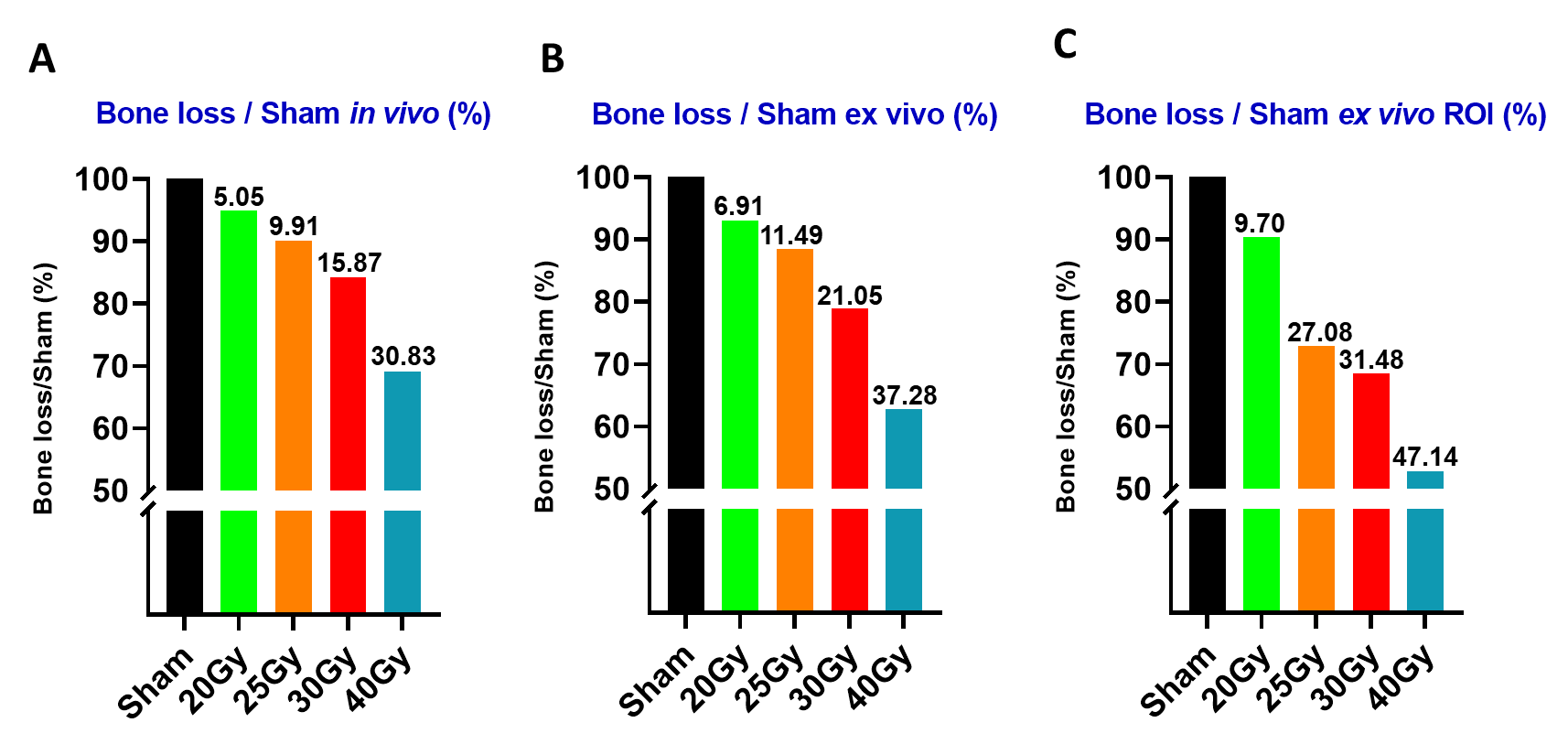
**

**Supplementary Figure 3. Graphics representing total mandibular (left) bone volume quantification according to irradiation doses at 8 W and 12 W (*in vivo* CT-images).** ns: not statistical, * versus sham, # versus groups. Statistical analysis: Shapiro normality test then parametric One way ANOVA.

**
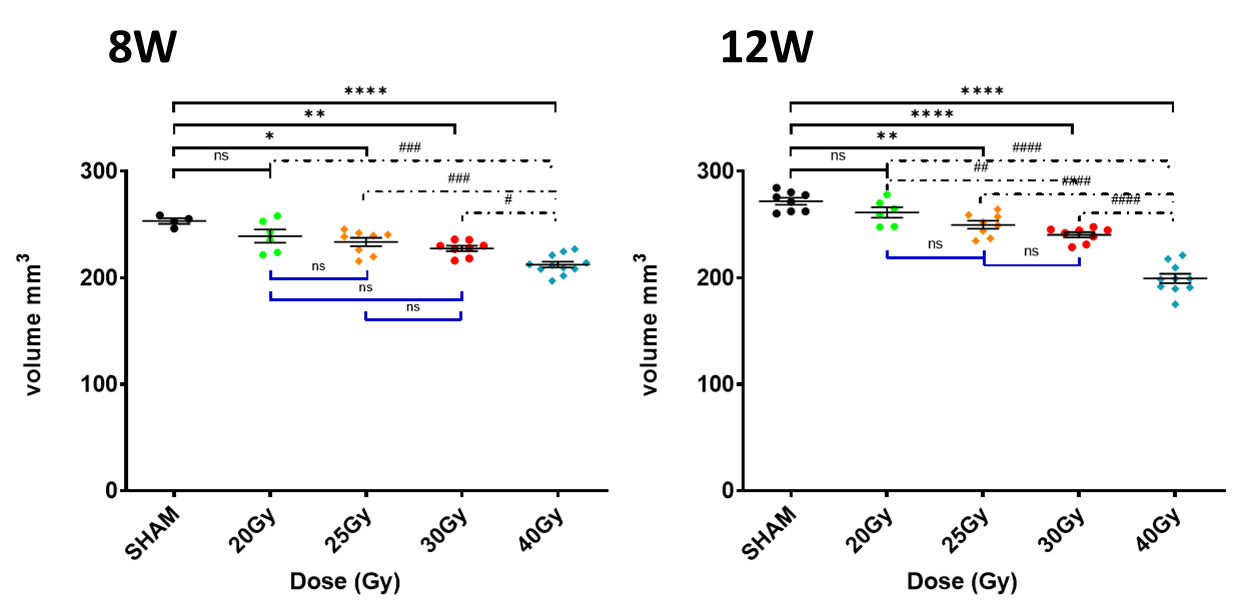
**
